# Impact of Severe Extracranial ICA Stenosis on MRI Perfusion and Diffusion Parameters in Acute Ischemic Stroke

**DOI:** 10.3389/fneur.2014.00254

**Published:** 2014-12-05

**Authors:** Philipp Kaesemann, Götz Thomalla, Bastian Cheng, Andras Treszl, Jens Fiehler, Nils Daniel Forkert

**Affiliations:** ^1^Department of Computational Neuroscience, University Medical Center Hamburg-Eppendorf, Hamburg, Germany; ^2^Department of Neurology, University Medical Center Hamburg-Eppendorf, Hamburg, Germany; ^3^Department of Medical Biometrics and Epidemiology, University Medical Center Hamburg-Eppendorf, Hamburg, Germany; ^4^Department of Diagnostic and Interventional Neuroradiology, University Medical Center Hamburg-Eppendorf, Hamburg, Germany

**Keywords:** magnetic resonance imaging, brain ischemia, internal carotid artery stenosis, perfusion, diffusion

## Abstract

**Purpose:** The aim of this study was to investigate the impact of a coexisting internal carotid artery (ICA) stenosis on lesion volumes as well as diffusion and perfusion parameters in acute ischemic stroke resulting from middle cerebral artery (MCA) occlusion.

**Material and methods:** Magnetic resonance imaging data of 32 patients with MCA occlusion with or without additional ICA stenosis imaged within 4.5 h of symptom onset were analyzed. Both groups consisted of 16 patients. Acute diffusion lesions were semi-automatically segmented in apparent diffusion coefficient (ADC) MRI datasets. Perfusion maps of cerebral blood volume (CBV), cerebral blood flow, mean transit time and *T*_max_ were calculated using perfusion-weighted MRI datasets. Tissue-at-risk (TAR) volumes were generated by subtracting the ADC lesion from the hypoperfusion lesion defined by *T*_max_ >6 s. Median ADC and perfusion parameter values were extracted separately for the diffusion lesion and TAR and used for statistical analysis.

**Results:** No significant differences were found between the groups regarding the diffusion lesion and TAR volumes. Statistical analysis of diffusion and perfusion parameters revealed CBV as the only parameter with a significant difference (*p* = 0.009) contributing a small effect (η^2^ = 0.11) to the group comparison with higher CBV values for the patient group with a coexisting ICA stenosis, while no significant effects were found for the other diffusion and perfusion parameters analyzed.

**Conclusion:** The results of this study suggest that a coexisting ICA stenosis does not have a strong effect on tissue status or perfusion parameters in acute stroke patients except for a moderate elevation of CBV. This may reflect improved collateral circulation or ischemic preconditioning in patients with a pre-existing proximal stenosis balancing impaired perfusion from the stenosis.

## Introduction

Multi-parametric magnetic resonance imaging (MRI) is nowadays widely available and frequently used in most stroke centers (1). In particular, perfusion-weighted MR imaging (PWI) and diffusion-weighted MR imaging (DWI) have become important tools for today’s diagnosis and treatment decision making in acute ischemic stroke patients.

Diffusion-weighted MR imaging is capable of displaying the water diffusion property of the cerebral tissue with high sensitivity (2). By this, DWI allows detecting acute ischemic stroke lesions within minutes after stroke symptom onset whereas brain regions displaying a strong diffusion restriction are assumed to represent the infarct core (3). PWI datasets on the other hand, enable the identification of cerebral tissue with reduced blood perfusion. It is typically assumed that the infarct core gradually expands into the hypoperfused tissue over time (4). The volumetric difference between the lesions defined in the PWI and DWI datasets is often used in the clinical and research setting as a surrogate for the ischemic penumbra or tissue-at-risk (TAR) (5–7). In general understanding, this TAR is the target for reperfusion therapy, e.g., using intravenous thrombolysis or thrombectomy, since it is assumed that the brain cells in this penumbra region are still salvageable in case of timely reperfusion.

The most common “phenotypic mechanisms” regarding the formation of an acute ischemic stroke are categorized by the TOAST (8) and CCS-classification system (9) into the four subtypes: large-artery atherosclerosis, cardioembolism, small-vessel occlusion, and strokes with undetermined etiology, which includes combinations of all aforementioned subtypes. Pathophysiological considerations identify plausible differences between acute strokes resulting from different etiologies regarding acute perfusion changes and collateral activation. Simplified, an acute embolic occlusion (cardioembolic stroke) is assumed to hit more or less healthy brain vasculature. In contrast to this, additional impairment of brain perfusion has to be considered in stroke resulting from large-artery atherosclerosis with pre-existing extracranial internal carotid artery (ICA) stenosis.

Thus, it may be hypothesized that an acute stroke patient with an artery occlusion and coexisting stenosis will suffer of a more serious hypoperfusion situation leading to a faster stroke evolution and worse outcome compared to a patient with the same artery occlusion but without a coexisting stenosis. On the other hand, it is widely accepted that the collateralization situation is a profound factor for the outcome severity after acute stroke (10, 11). Within this context, it may be hypothesized that a chronic carotid stenosis leads to a more profound development of the collateral circulation to compensate the long-lasting and constant perfusion impairment, which may be even beneficial to endure a hypoperfusion in case of an acute ischemic stroke. Another possible result of the perfusion deficit caused by a chronic carotid stenosis could be an ischemic preconditioning of the brain tissue leading to a protective effect as already described for cardial (12) and cerebral tissue (13).

The aim of this study was to study the impact of pre-existing high-grade ICA stenosis on tissue pathology in acute stroke. Within this context, we tested for differences in diffusion and perfusion parameters as well as volumes of infarct core and TAR of infarction between patients with occlusion of the proximal middle cerebral artery (MCA) with and without coexisting unilateral stenosis of the extracranial ICA.

## Materials and Methods

### Patients

In this retrospective cohort study, we examined MRI datasets of 32 patients admitted to our hospital with acute ischemic stroke due to occlusion of the MCA main stem alone (M1) or with coexisting ipsilateral stenosis of the ICA (M1 + ICA). Overall, 16 consecutive cases of M1 occlusion with coexisting ICA stenosis were included in this study, while 16 non-consecutive patients with M1 occlusion but without ICA stenosis were selected from our local database with a focus on achieving a balanced and age-matched group for comparison. The ICA stenosis was diagnosed by ultrasound imaging during the next 5 days after stroke symptom onset according to the North American Symptomatic Carotid Endarterectomy Trial (NASCET) criteria (14) with a threshold >70%. A stenosis above this threshold is usually considered to represent a “high grade” and, thus, hemodynamically relevant ICA stenosis, which is also in accordance with consensus criteria established by expert panels of radiologists (15, 16). Patients with complete ICA stenosis (100%) were excluded from this study. After an initial exclusion of an intracerebral bleeding, PWI and DWI datasets were acquired among other MR image sequences within 4.5 h after symptom onset and prior to treatment in all patients.

The study was approved by the local ethics committee and informed consent was obtained from all subjects.

### MR imaging

All MRI measurements were performed on a 1.5 T Avanto Scanner (Siemens, Erlangen, Germany).

Gradient-echo dynamic susceptibility contrast PWI datasets were acquired over a period of 90 s after application of contrast agent (approximately 15 ml of Bayer Magnevist, Bayer HealthCare, Leverkusen, Germany) with a TR = 2000 ms, TE = 45 ms, and flip angle = 90° and an image in-plane resolution of 0.94 mm^2^. Each PWI dataset consisted of 24 slices of 5 mm thickness.

For DWI acquisition, a TR = 3500 ms, TE = 89 ms, flip angle = 90°, and diffusion weightings of 0 and 1000 s/mm^2^ measured in three directions (in *x, y*, and *z* direction) were used. The image in-plane resolution and slice thickness of the DWI datasets were equal to the PWI datasets.

### Data analysis

In time-resolved 3D dynamic susceptibility contrast PWI datasets, the passage of contrast agent results in a reduction of signal values due to shortening of relaxation times. To enable a quantitative perfusion analysis, the single data points of each temporal signal curve *S*(*t*) in the PWI datasets need to be converted to relative transverse relaxivity $\Delta R_{2}^{*}\left(t\right)$ changes using the following equation:

(1) $$\Delta R_{2}^{*}\left(t\right)=-\frac{k}{\mathrm{TE}}\ln\left(\frac{S\left(t\right)}{S_{0}}\right)$$

where *k* denotes a proportionality constant, which was set to 1, TE the echo time, and *S*_0_ the baseline MRI signal. Since $\Delta R_{2}^{*}\left(t\right)$ is only relative to the real contrast agent concentration, the correction formulas and values described in Ref. (17) were used for conversion of $\Delta R_{2}^{*}\left(t\right)$ to concentration time curves *C*(*t*) in which the time-dependent contrast agent concentration is measured in millimoles:

(2) $$\Delta R_{2}^{*}\left(t\right)=r_{\mathrm{tissue}}C\left(t\right),$$

where *C* is the contrast agent concentration and *r*_tissue_ the relaxation effect, which was set to 0.044 ms × mM^−1^ as appropriate for 1.5 T field strength. After conversion to concentration time curves, quantitative perfusion parameters were calculated for each voxel of the PWI dataset by solving the following equation:

(3) $$C\left(t\right)=\mathrm{CBF}\cdot R\left(t\right)⊗C_{a}\left(t\right),$$

where *C*(*t*) denotes the tissue concentration curve, CBF the cerebral blood flow, *C*_a_(*t*) the arterial input function, *R*(*t*) the residue function, and ⊗ the convolution operator. The arterial input function was interactively selected by an experienced observer as a single voxel next to the proximal segment of the MCA contralateral to the diffusion deficit with dynamic visualization of the concentration time curves. Afterward, the selected AIF for each patient was corrected using the corresponding correction formula and parameters for large vessels described in Ref. (17):

(4) $$\Delta R_{2}^{*}\left(t\right)=\mathrm{rC}\left(t\right)+\mathrm{qC}{\left(t\right)}^{2},$$

Here, *r* = 7.6 × 10^−3^ ms × mM^−1^ and *q* = 574 × 10^−6^ ms × mM^2^ were used for correction of the AIFs obtained from the 1.5 T PWI datasets.

The block-circulant singular value decomposition as described by Wu et al. (18) together with a truncation threshold of 0.2 was used to solve the Eq. (2). Apart from the CBF parameter, which results directly from the deconvolution in terms of the maximum value of the residual function, the cerebral blood volume (CBV) parameter was determined by calculating the area under the residue function, and the *T*_max_ parameter as the time point where the residue function reaches its maximum. Finally, the mean transit time (MTT) parameter was calculated according to the central volume theorem (19) dividing the CBV by the CBF parameter.

For segmentation of the acute diffusion restriction, the DWI dataset acquired with the two different *b*-values was used to calculate the corresponding apparent diffusion coefficient (ADC) parameter map using the Stejskal–Tanner equation (20). The DWI lesion was then segmented by interactively placing at least one seed point within the diffusion restriction, which was then used within a volume growing segmentation with a fixed upper ADC-threshold of 550 × 10^9^ mm^2^/s (21, 22).

**Figure 1:**
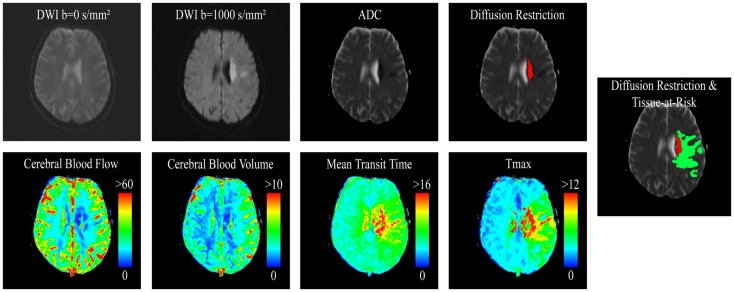
**Selected slice from a DWI dataset and corresponding apparent diffusion coefficient (ADC) parameter dataset used for definition of the diffusion restriction as well as corresponding perfusion parameter maps CBF (in milliliter per 100 gram per second), CBV (in milliliter per 100 gram), MTT (in seconds), and *T*_max_ (in seconds), also used for tissue-at-risk definition**.

For definition of the hypoperfused tissue, the PWI parameter maps were registered to the DWI sequences using a rigid registration technique with linear interpolation and maximization of the mutual information metric. After this, the segmented voxels of the diffusion restriction were used as seeds for a second volume growing segmentation based on the *T*_max_ maps. In accordance to recent stroke studies (23), a threshold of *T*_max_ >6 s was used for the definition of hypoperfused tissue. The DWI lesion was then subtracted from the perfusion lesion to obtain two volumes-of-interests (VOIs), which do not overlap. These two VOIs (DWI lesion and TAR) were used for lesion volume quantification as well as extraction of the corresponding median values of ADC and four perfusion parameters (*T*_max_, MTT, CBV, and CBF), which were used for statistical analysis (see Figure 1). Median instead of average values were used in this study due to the non-normal distribution of the physiological parameters.

All image processing steps were performed in this work using the in-house developed software tool AnToNIa (7).

### Statistical analysis

The distribution of each parameter was examined. In case of skew distributions, the median and interquartile range (IQR) are reported and in case of a normal distribution, the mean and corresponding standard deviation are reported.

Prior to performing statistical tests, the continuous values of the five perfusion and diffusion parameters were transformed to normal distributions using the Box–Cox technique (24), if required. Group comparisons were performed using Student’s *t*-test for variables in interval scale with a normal distribution, Mann–Whitney *U*-test for variables in interval scale without normal distribution, and Fisher’s-exact-test for variables in nominal scale.

A two-way MANOVA was used to compare the diffusion and perfusion parameters for region (DWI lesion vs. TAR) and group (M1 vs. M1 + ICA). All tests were performed two-sided. A *p*-value <0.05 was considered significant. Nominal *p*-values are reported without correction for multiplicity.

For the interpretation of effect sizes (25), a partial squared η ≥ 0.04 was considered as a small, a partial squared η ≥ 0.25 as a moderate, and a partial squared η ≥ 0.64 as a large effect in accordance with the recommendations by Ferguson (26).

The statistical analysis was performed using IBM SPSS Statistics (Version 19.0, IBM, Armonk, NY, USA).

## Results

### Clinical characteristics

Clinical characteristics of the patients included in this retrospective study are given in Table 1. Mean age was similar in both groups (68 years for the M1 group and 64 years in the M1 + ICA group). In contrast to this finding, there was an imbalance regarding the gender distribution with 81.3% female patients in the M1 group compared to only 18.8% female patients in the M1 + ICA group. Mean NIHSS-score at admission was 15 points in both groups. Time from symptom onset to MRI was also comparable for both groups.

**Table 1:** **Patient’s baseline data (mean ± SD)**.

	M1	M1 + ICA	*p*-Value
**Gender**		
Male	3 (18.8%)	13 (81.3%)	0.001
Female	13 (81.3%)	3 (18.8%)	0.001
Age (years)	68 (±12)	64 (±13)	0.328
NIHSS at admission	15 (±5)	15 (±6)	0.780
Time-to-MRI (minutes)	189 (±94)	137 (±65)	0.145

### Infarct lesion volumes and perfusion parameters

Overall, large TAR volumes were found in both groups (see Table 2). Both the DWI lesion and the TAR volume were larger in the M1 group. However, this difference was not significant.

**Table 2:** **Median lesion volumes and interquartile range (Q1–Q3)**.

Region	M1	M1 + ICA	*p*-Value
DWI-lesion volume (ml)	9.7 (2.7–17.1)	4.8 (1.7–8.6)	0.381
Tissue-at-risk volume (ml)	69.0 (42.9–100.2)	52.2 (29.5–106.6)	0.724

The results of the quantitative perfusion and diffusion analysis are given in Table 3. With respect to the different lesion compartments, lower median ADC, CBF, and CBV values were found in the DWI lesion compared to the TAR volume independent of the group, while prolonged median *T*_max_ values were found in the DWI lesion. Regarding the comparison of patients groups, the median ADC, CBF, CBV, and *T*_max_ values appeared elevated in both lesion compartments in the group with coexisting ICA stenosis. The overall highest relative difference between the groups was found for the CBV parameter regardless of the lesion component and CBF parameter within the DWI lesion.

**Table 3:** **Median VOI-based diffusion and perfusion parameters and interquartile range (Q1–Q3)**.

Parameter	M1	M1 + ICA
**DWI lesion**	
ADC (10^−6^ mm^2^/s)	467.8 (453.3–531.6)	482.3 (445.2–525.4)
*T*_max_ (s)	14.1 (11.9–16.7)	15.1 (12.5–19.4)
CBF (ml/100 g/min)	10.9 (7.3–13.7)	14.0 (8.3–22.5)
CBV (ml/100 g)	2.1 (1.5–2.6)	2.7 (1.9–5.0)
MTT (s)	13.9 (11.5–14.8)	13.9 (11.7–16.4)
**Tissue-at-risk**	
ADC (10^−6^ mm^2^/s)	824.1 (775.1–864.9)	839.8 (825.0–863.1)
*T*_max_ (s)	11.2 (9.7–12.9)	12.1 (10.4–14.2)
CBF (ml/100 g/min)	20.7 (15.6–24.0)	21.9 (15.5–27.3)
CBV (ml/100 g)	3.5 (3.2–4.4)	4.2 (3.1–5.9)
MTT (s)	11.7 (11.1–13.5)	13.9 (12.5–14.8)

MANOVA analysis revealed four parameters with a significant difference and notable effect size regarding the two lesion compartments (see Table 4). More precisely, the difference in ADC values between the DWI lesion and TAR showed a η^2^ = 0.90 (*p* = 0.001), which denotes a large effect. However, since thresholding of the ADC parameter map was used for definition of the DWI lesion, this difference is not surprising. Furthermore, significant differences and small effect sizes between the two lesion compartments were also found for the *T*_max_ (*p* = 0.003, η^2^ = 0.14), CBF (*p* = 0.002, η^2^ = 0.15), and CBV (*p* = 0.014, η^2^ = 0.10) perfusion parameters.

**Table 4:** **Diffusion and perfusion parameter effect sizes**.

Parameter	Lesion compartment (DWI vs. TAR)	Group (M1 vs. M1 + ICA)
ADC	**η^2^** **= 0.90 (*p* = 0.001)**	η^2^ = 0.03 (*p* = 0.150)
*T*_max_	**η^2^** **= 0.14 (*p* = 0.003)**	η^2^ = 0.01 (*p* = 0.440)
CBF	**η^2^** **= 0.15 (*p* = 0.002)**	η^2^ = 0.03 (*p* = 0.149)
CBV	**η^2^** **= 0.10 (*p* = 0.014)**	**η^2^ = 0.11 (*p* = 0.009)**
MTT	η^2^ = 0.02 (*p* = 0.305)	η^2^ = 0.04 (*p* = 0.108)

*Bold font indicates p < 0.05*.

Regarding the comparison of the two patient groups, a significant difference (*p* = 0.009) and a notable effect size of η^2^ = 0.11 was only found for the CBV perfusion parameter. In other words, a pre-existing carotid stenosis explains 11% of the variance in CBV. Apart from this, all other parameters tested in this study did not reach significance in the MANOVA analysis.

## Discussion

In our comparison of diffusion and perfusion parameters, as well as lesion volumes in acute ischemic stroke, the CBV was the only perfusion parameter with a considerable difference between patients with and without coexisting high-grade stenosis of the ICA. More precisely, CBV was significantly higher in patients with a M1 + ICA stroke, which holds true for the DWI lesion as well as TAR. However, the effect of pre-existing ICA stenosis on CBV was rather small. All other diffusion and perfusion parameters tested in this study contributed no significant effect to the group comparison. To sum up, this finding relativizes speculations on the impact of large-artery atherosclerosis on brain perfusion in acute ischemic stroke.

In the setting of carotid stenosis in patients without stroke, Lythgoe et al. (27) studied MRI perfusion parameters (CBF, CBV, and MTT) in 16 patients with unilateral carotid stenosis and compared the findings with PET data. Overall, a mean CBF value of 43 ml/100 g/min, a mean CBV value of 3.8 ml/100 g, and a mean MTT value of 6.2 s was found for the analyzed group of patients with unilateral stenosis. In our study, we found considerably smaller CBF, longer MTT, and slightly smaller CBV values. These considerable differences are most likely an expression of the concomitant acute stroke. Lythgoe et al. concluded from the results of their study that the extent of the MTT prolongation corresponds best to the degree of the carotid stenosis, whereas the change in CBF and CBV could indicate hemodynamic alterations due to compensatory collateralization. In agreement with our findings, an average increase in CBV values was observed, while a CBF decrease was only found in few patients.

In a similar study by Kluytmans et al. (28), which focused on the comparison of MRI perfusion parameters between gray and white matter in 17 patients with symptomatic unilateral carotid stenosis without stroke and a control group, no significant alteration of the CBF values but a significant increase of CBV and MTT values was found in the stenosis group. Nighoghossian et al. (29) published a similar work including five patients with symptomatic unilateral carotid stenosis also without an acute stroke, in which prolonged MTT and higher CBV values compared to normal subjects were found. This is similar to the slightly increased CBV in acute stroke patients with coexisting ICA stenosis as found in our study.

So far, there is only limited data regarding the diffusion and perfusion situation in case of an acute MCA occlusion and concurrent carotid stenosis. For example, Neumann-Haefelin et al. (30) conducted a study investigating MRI perfusion parameters [TTP, MTT, relative CBV (rCBV) and relative CBF (rCBF)] and TAR volume in 28 stroke patients before and after intravenous thrombolysis. The results of this study revealed significantly larger TAR volumes in the stenosis group while the resulting infarct lesions in the follow-up MRI datasets were significantly smaller compared to the group with MCA occlusion without stenosis. Regarding the MRI perfusion parameters, no significant differences were found for ADC, TTP, MTT, rCBV, and rCBV values in the DWI lesion or in the TAR volume. Significantly higher TTP and MTT and significantly lower rCBF were only found in the peripheral regions of the TAR for the subgroup with carotid stenosis. In contrast to our analysis, in which absolute CBF and CBV values were used, the relative CBF as a parameter adjusted to the contralateral unaffected hemisphere was calculated. Absolute perfusion values were used in this study since it was previously demonstrated that a stenosis may not only result in perfusion changes in the affected hemisphere but also in the contralateral hemisphere (31) such that a normalization with average perfusion values from the contralateral hemisphere may lead to erroneous results. On the contrary, it needs to be emphasized that absolute perfusion values as used in this work are dependent on the selected arterial input function to a higher degree than relative perfusion values. However, great care was taken in this study to select good and comparable arterial input functions such that the influence of this should be less important.

Another reason for the different results found in this study compared to the study by Neumann-Haefelin et al. may be the type of deconvolution technique used for perfusion parameter map calculation. More precisely, it has been shown that the block-circulant singular value decomposition, which was used in this work, is less sensitive to contrast agent arrival time differences between the arterial input function and tissue compared to the standard singular value decomposition (18) as used in the study by Neumann-Haefelin et al. (30). Within this context, it has been shown that the *T*_max_ (32) as well as the CBF parameter (18) are especially affected by the simple contrast agent delay when calculated using a standard singular value decomposition. Overall, the results of this study suggest that the impact of an additional ICA stenosis on typically used perfusion parameters is rather low, such that common thresholds used for perfusion-based stroke analysis and quantification may even be valid in case of a coexisting ICA stenosis.

Based on simple pathophysiological considerations, a pre-existing severe internal carotid stenosis may limit the capability to compensate an acute MCA occlusion event by reduced perfusion pressure onto the occluded artery and by reduced blood flow via collaterals supplied by the anterior cerebral artery. This should be measurable using MRI perfusion parameters in terms of a prolonged MTT and *T*_max_ as well as a decreased CBF and CBV. One might also expect larger stroke lesion volumes in patients with an additional carotid stenosis. However, the results of our study do not support this assumption since the CBV perfusion parameter was the only parameter for which a significant difference reaching only a small effect size was found in the group comparison, with slightly elevated values in the ICA + M1 group. Likewise, no significant differences were found regarding the volumes of the DWI lesion or TAR between the two groups.

One possible explanation for these findings may be that a carotid stenosis, which was developed over a long time, may lead to the development of cerebral collateralization as a compensatory mechanism, for which Lythgoe et al. found some evidence in their study in terms of elevated CBV values (27). Within this context, it was, for example, demonstrated by Kim et al. (11) that the status of the collateralization does not only affect the cerebral hemodynamics, but also the clinical outcome. Furthermore, Bang et al. (10) found that the presence of collateral flow improves the response toward intravenous thrombolysis. This implicates that collateralization plays an important clinical role in acute ischemic stroke.

The main finding of this study that the diffusion and perfusion differences between the two groups are rather subtle except for the CBV parameter may be an indication that the collateral situation is indeed improved in these patients. A recent study by Cortijo et al. (33) suggests that an increased CBV is the best perfusion marker for a good collateral situation. Thus, the fact that the CBV is the only parameter in this study for which a considerable alteration between the two groups was found further supports the theory of an improved collateral circulation in patients with a coexisting stenosis. The finding that no significant differences were found between the two groups regarding the DWI lesion as well as TAR volume may be also an indicator for an improved collateral circulation in patients with a coexisting stenosis. However, no systematically collected data on collateral activation were available for this retrospective study such that this point remains speculative.

Another potential reason for the elevated CBV values as well as the similar DWI and TAR volumes found in this study could be an ischemic preconditioning caused by the coexisting carotid stenosis. A study by Wegener et al. (13) investigated MRI datasets (DWI datasets) of 65 patients with a first-time occurrence of an ischemic territorial stroke with and without prodromal transient ischemic attacks (TIA). In brief, significantly smaller infarct lesions (as defined in ADC parameter maps) were found in patients who had a prodromal transient ischemic attack while the analyzed perfusion parameters (MTT, CBF, CBV) were similar in both groups. In our study, only a non-significant trend for smaller DWI-lesion volumes as well as increased CBV values was found in the stenosis group. This may result from the fact, that a previous TIA and pre-existing ICA stenosis do not have the same ischemic preconditioning effect, and it may be hypothesized that brain perfusion is more strongly compromised in a symptomatic TIA compared to an asymptomatic carotid stenosis. On the other hand, severe hypoperfusion originating from a stenosis affecting the same brain region as a subsequent stroke could induce ischemic preconditioning due to additional recruitment of local mechanisms like nitric oxide (34). Within this context, it was, for example, found in preclinical studies that an effective ischemic preconditioning can be achieved after temporal occlusion of the MCA in mice (35).

There are limitations to this study. First, the number of datasets used in this retrospective study is small and results need to be considered exploratory. Second, we were not able to analyze final infarct volumes as follow-up datasets were not available for all patients included in this study. Third, the precise stenosis degree was not available anymore for all patients but only the graded information, e.g., high grade >70% as used in this study as an inclusion criterion. Thus, it was not possible to correlate the perfusion, diffusion, and volumetric metrics to the precise stenosis degree, which would be an interesting subject for future studies. It should be also highlighted that patients with a complete stenosis (100%) were excluded from this study since it was hypothesized that the effect of a complete ICA occlusion on perfusion, diffusion, and lesion volumes may differ considerably from that of a high grade but non-complete stenosis. Thus, the inclusion of patients with a complete stenosis might lead to a more heterogeneous study group. However, this hypothesis should be tested in more detail in further studies. Finally, no differentiation of white and gray matter, which may be relevant for the CBF and CBV perfusion parameter, was performed since high-resolution T1-weighted MRI datasets were not available due to MR acquisition time limitations.

In summary, the results of our study suggest that extracranial ICA stenosis does not have a strong effect on brain perfusion in case of an acute ischemic stroke except for slightly elevated CBV values. This may indicate improved collateral circulation or ischemic preconditioning resulting from chronic hypoperfusion, which counteract the additional perfusion impairment in acute stroke patients with a coexisting stenosis.

## Conflict of Interest Statement

The Review Editor Jan Sobesky, declares that, despite having collaborated with authors Götz Thomalla, Jens Fiehler and Nils Daniel Forkert, the review process was handled objectively and no conflict of interest exists. The authors declare that the research was conducted in the absence of any commercial or financial relationships that could be construed as a potential conflict of interest.
